# Potential influencing factors of aortic diameter at specific segments in population with cardiovascular risk

**DOI:** 10.1186/s12872-022-02479-y

**Published:** 2022-02-05

**Authors:** Tingting Chen, Xingan Yang, Xiaoxin Fang, Lijiang Tang, Yang Zhang, Yingzheng Weng, Hongliang Zhang, Juntao Wu, Ping Mao, Baohui Xu, Jianjun Jiang, Xiaofeng Chen

**Affiliations:** 1 Department of Cardiology, Zhejiang Hospital, Hangzhou, 310013 Zhejiang Province China; 2 Department of Cardiology, Taizhou Hospital Affiliated to Wenzhou Medical University, Linhai, 317000 Zhejiang Province China; 3 Laboratory of Cardiovascular Disease, Taizhou Hospital Affiliated to Wenzhou Medical University, Linhai, 317000 Zhejiang Province China; 4 Department of Ultrasonic, Taizhou Hospital Affiliated to Wenzhou Medical University, Linhai, 317000 Zhejiang Province China; 5 Department of Cardiology, Taizhou Hospital, Zhejiang University School of Medicine, Hangzhou, 310013 Zhejiang Province China; 6 Department of Surgery, Stanford University School of Medicine, Stanford, CA 94305 USA; 7 Department of Radiation Oncology, Indiana University School of Medicine, Indianapolis, IN 46202 USA

**Keywords:** Aortic diameter, Aorta dilation, Risk factors, Ultrasonography

## Abstract

**Background:**

Aortic diameter is a critical parameter for the diagnosis of aortic dilated diseases. Aortic dilation has some common risk factors with cardiovascular diseases. This study aimed to investigate potential influence of traditional cardiovascular risk factors and the measures of subclinical atherosclerosis on aortic diameter of specific segments among adults.

**Methods:**

Four hundred and eight patients with cardiovascular risk factors were prospectively recruited in the observational study. Comprehensive transthoracic M-mode, 2-dimensional Doppler echocardiographic studies were performed using commercial and clinical diagnostic ultrasonography techniques. The aortic dimensions were assessed at different levels: (1) the annulus, (2) the mid-point of the sinuses of Valsalva, (3) the sinotubular junction, (4) the ascending aorta at the level of its largest diameter, (5) the transverse arch (including proximal arch, mid arch, distal arch), (6) the descending aorta posterior to the left atrium, and (7) the abdominal aorta just distal to the origin of the renal arteries. Multivariable linear regression analysis was used for evaluating aortic diameter-related risk factors, including common cardiovascular risk factors, co-morbidities, subclinical atherosclerosis, lipid profile, and hematological parameters.

**Results:**

Significant univariate relations were found between aortic diameter of different levels and most traditional cardiovascular risk factors. Carotid intima-media thickness was significantly correlated with diameter of descending and abdominal aorta. Multivariate linear regression showed potential effects of age, sex, body surface area and some other cardiovascular risk factors on aortic diameter enlargement. Among them, high-density lipoprotein cholesterol had a significantly positive effect on the diameter of ascending and abdominal aorta. Diastolic blood pressure was observed for the positive associations with diameters of five thoracic aortic segments, while systolic blood pressure was only independently related to mid arch diameter.

**Conclusion:**

Aortic segmental diameters were associated with diastolic blood pressure, high-density lipoprotein cholesterol, atherosclerosis diseases and other traditional cardiovascular risk factors, and some determinants still need to be clarified for a better understanding of aortic dilation diseases.

## Background

Aorta dilation is a common problem in clinical practice, and the subsequent aortic aneurysm is a significant cause of adult death. The pathogenesis of aortic dilation is characterized by aortic wall inflammation, induction of smooth muscle cell apoptosis, extracellular matrix degradation, plaque formation, oxidative stress and vascular remodeling [1]. Potential biological mechanisms still need to be explored.

Aortic diameter is a critical parameter for the diagnosis of aortic aneurysms. The risk of rupture increases as the diameter of the aneurysm increases. Increased baseline diameter of infrarenal aorta is an independent risk factor for abdominal aortic aneurysm (AAA) in a population-based follow-up study [2]. Increased aortic aneurysm diameter is associated with a significantly increased risk of future cardiovascular events and all-cause mortality [3], while previous follow-up research holds the same view for the whole range of diameter values [4]. Therefore, exploring the risk factors for aortic dilation has great significance in predicting, preventing and treating cardiovascular disease.

Currently, research on the epidemiology of aortic diameter is gradually emerging. Many studies pointed out that aortic diameter and cardiovascular diseases shared common influencing factors. Age, sex, race, body surface area (BSA), smoking status, alcohol consumption, and lipid profile, hypertension and diabetes mellitus (DM) are all known major traditional cardiovascular risk factors which have effects on aortic diameter [5–8]. Atherosclerosis is well-known as one of the main causes of aneurysm. AAA prevalence is significantly higher among patients with coronary artery disease (CAD) [9]. Limited research suggests that subclinical atherosclerosis and carotid atherosclerosis are positively related to aortic diameter growth [10, 11]. However, there are inconsistent views among the current studies concerning the risk factors that affect the aorta diameter. Also, the segment-specific difference in the relationship between risk factors and aortic diameter has been indicated by previous research under different experimental conditions [12, 13]. However, related research incorporated a small number of cardiovascular indicators and evaluated fewer aorta segments.

In the current study, in order to further explore the mechanism of the early stage of aortic dilation, we examined the association between nine segmental aortic diameters of non-aneurysmal aortas and cardiovascular risk factors and the measures of subclinical atherosclerosis.

## Methods

### Study population

A total of 520 consecutive hospitalized patients with cardiovascular risk factors were admitted from Taizhou Hospital affiliated to Wenzhou Medical University in China between December 2013 and December 2014 in the single-center observational study. Exclusion criteria were as follows: Marfan syndrome, aortic stenosis or aortic regurgitation more than mild in degree, active cancer, infective endocarditis, syphilitic aortitis, rheumatic heart disease, and valvular heart disease (n = 17). In addition, we excluded patients who couldn’t clearly show the long axis of the aorta due to poor image quality (n = 12) and patients with missing essential data (n = 83). Of the remaining, 408 patients participated in the final analysis. The study was in accordance with the Helsinki Declaration and approved by the Institutional Review Board. Informed consent was obtained from all participants.

### Baseline characteristic collection

Information on the variables of interest like the patient's history of CAD, DM, hypertension, dyslipidemia, chronic obstructive pulmonary disease (COPD), cerebral infarction, medication history, and status of drinking and smoking could be collected after admission. Hypertension was defined as twice or more measured systolic blood pressure (SBP) ≥ 140 mmHg or diastolic blood pressure (DBP) ≥ 90 mmHg measured at rest on the same day or current use of antihypertensive agents. DM was defined as currently using physician-prescribed hypoglycemic drugs or fasting serum glucose levels > 7 mmol/L and postprandial blood glucose > 11.1 mmol/L. CAD referred to current or previous history of typical angina, acute coronary syndrome, or meeting the gold standard that detected coronary artery occlusion or stenosis by the coronary angiography technique. Hyperlipidemia was previously diagnosed with the plasma total cholesterol > 6.5 mmol/L or plasma triglyceride level > 1.7 mmol/L or current use of lipid-lowering therapy. Alcohol and smoking status were defined as YES or NO. Carotid intima-media thickness (CIMT), carotid atherosclerosis, carotid artery stenosis, and aortic sclerosis were detected by ultrasonography technique. SBP and DBP were measured on the seated subject’s right arm in a calm state. The average of the second and third measurements was recorded. BSA was calculated according to the Stevenson formula: BSA(m^2^) = 0.0061 × height (cm) + 0.0128 × weight (kg) − 0.1529.

All patients received venous blood withdrawal after fasting overnight. Serum levels of total cholesterol (TC), high-density lipoprotein cholesterol (HDL-C), low-density lipoprotein (LDL-C), apolipoprotein A1, apolipoprotein B, lipoprotein (a), triglyceride, C-reactive protein (CRP), and hematological parameters including white blood cell (WBC) count, red blood cell (RBC) count, hemoglobin and platelet count, were detected in the clinic laboratory by commercially available standardized methods.

### Evaluation of the aorta diameter by ultrasonography technique

Comprehensive transthoracic M-mode, 2-dimensional Doppler echocardiographic studies were performed using a commercial and clinical diagnostic ultrasonography technique. Doppler echocardiographic examination was conducted on a GE Sonoline echocardiograph (GE, vivid E9, Boston, MA, USA) with a variable frequency probe from 2.5 to 5.5 MHz, and operated in strict accordance with the guidelines of the echocardiograph. The aortic dimensions were assessed at different levels: (1) the annulus, (2) the mid-point of the sinuses of Valsalva, (3) the sinotubular junction, (4) the ascending aorta at the level of its largest diameter, (5) the transverse arch including proximal arch, mid arch and distal arch, (6) the descending aorta posterior to the left atrium, and (7) the abdominal aorta just distal to the origin of the renal arteries. The aortic diameter was measured using the inner-inner line method, and the largest diameter was reported. The aortic root and the ascending aorta were taken on the parasternal long-axis view. The aortic arch measurement was taken on the superior sternal fossa long-axis view. The descending aorta was measured along the short-axis of the left ventricle next to the sternum. The abdominal aorta was measured along the long axis under the xiphoid process [14]. Except for the measurement of the aortic annulus in the mid-systole, the rest of the aortic segments were measured in the end-diastole, making sure that the measurement line was perpendicular to the long axis of the aorta. Measurements were averaged from 3 to 5 beats. The measurement was completed by two professional technicians with more than five years of work experience and the measurement data was completed by one of the technicians.

### Statistical analyses

Continuous variables that conformed to normal distribution were expressed as mean values, and its standard deviation, and that non-normally distributed the median and its range represented variables. Categorical variables were expressed in percentiles. Differences between the two groups were assessed using independent samples T-test or Mann–Whitney U-test for continuous variables according to whether the data obeys normality distribution. The chi-square test was for categorical variables. Baseline variables (including sex, age, BSA, smoking, drinking, SBP, DBP, hypertension, DM, CAD, hyperlipidaemia, triglyceride, HDL-C, LP (a), statin agent, hypoglycemic agent and anti-hypertension agent) that were considered clinically relevant or showed a univariate relationship with the aortic diameter with *P* < 0.1 were entered into multivariate linear regression analysis. Variables for inclusion were carefully chosen, given the number of events available, to ensure the parsimony of the final models. An unstandardized regression coefficient was used to represent an increase or decrease in the outcome as the variables change. Variance inflation factor (VIF) was applied to evaluate the collinearity in the multiple regression model. Variables with a VIF ≥ 3, the index for the presence of collinearity, have been excluded from our analysis, including TC, LDL-C, apolipoprotein A1 and apolipoprotein B. Values of *P* < 0.05 were considered statistically significant. Data analysis and processing were performed by SPSS (version 20.0) package.

## Results

### Baseline characteristics

**Fig. 1:**
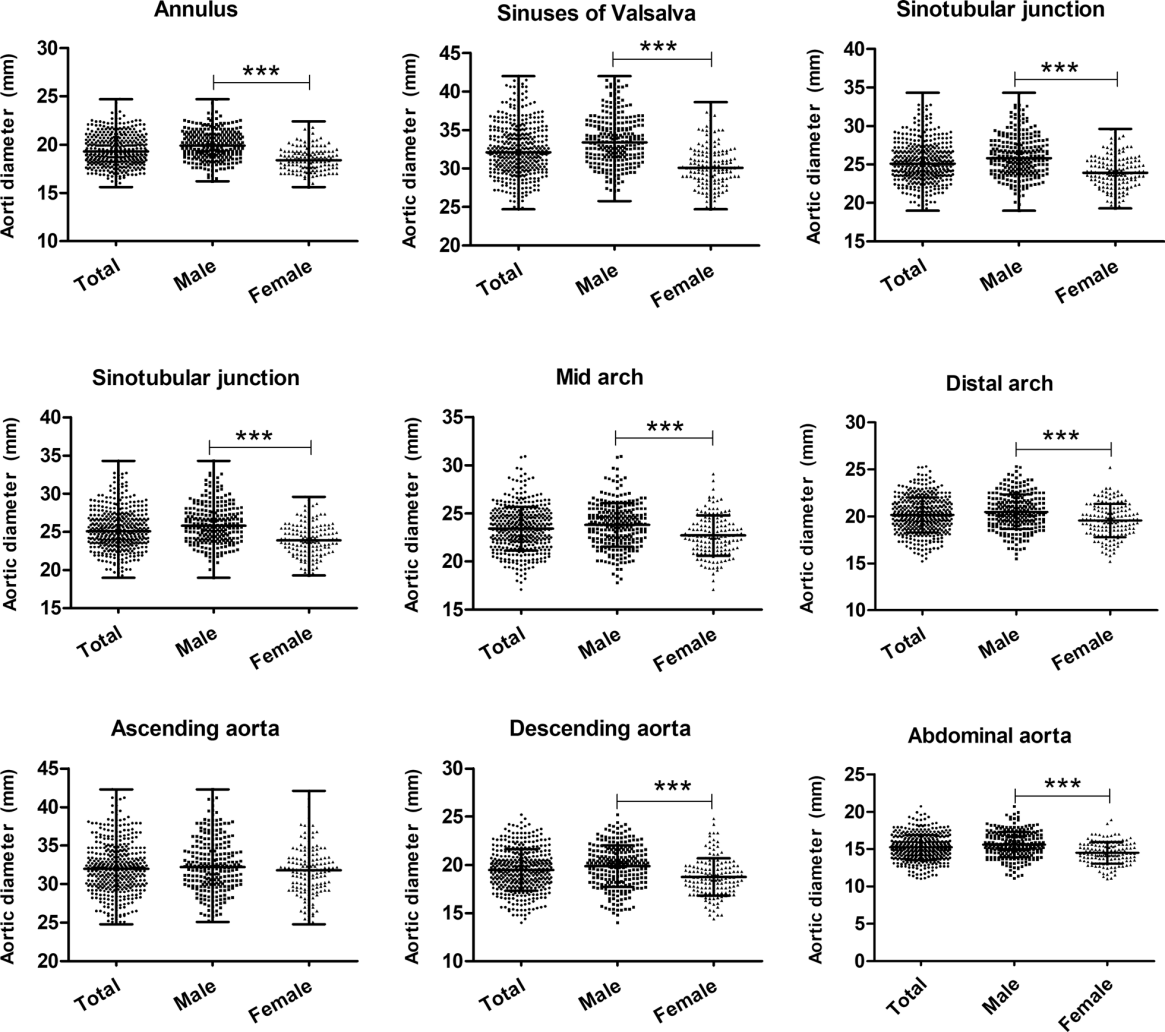
Sex difference in each level of aortic dimension

Consecutive 408 patients were analyzed in the study. The average age of participants was median 66 years (range from 40 to 92 years old), and 36% (147) were female. The data (Table 1) showed no difference in age, DBP, serum levels of lipoprotein(a) and CRP, WBC count, use of antiplatelet, statins and antihypertensive agents, as well as the percentage of DM, hyperlipidaemia, carotid atherosclerosis, carotid artery stenosis and aortic sclerosis, between male and female groups. Meanwhile, SBP, serum lipid profile levels including TC, triglyceride, HDL-C, LDL-C and platelet count in females, were significantly higher than those in males. On the contrary, BSA, RBC count, hemoglobin and CIMT, alcohol and smoking status, use of hypoglycemic agents, as well as the presence of CAD, hypertension, cerebral infarction and COPD in males were greater than those in females. The aortic diameter of each segment was reported in Table 2. Almost all aortic segment diameters are significantly larger in the male group than in the female group, except for ascending aorta (*P* = 0.078), as illustrated in Fig. 1.

**Table 1:** Characteristic of participants

Variables	All Mean ± SD or median or n (%)	Male Mean ± SD or median or n (%)	Female Mean ± SD or median or n (%)	*P* value
Age, year	66 (40–92)	66 (40–91)	68 (41–92)	0.173
BSA	1.65 (1.32–2.14)	1.70 (1.35–2.14)	1.52 (1.32–1.96)	0.000
Smoking, n (%)	161 (39.5%)	157 (60.2%)	4 (2.7%)	0.000
Alcohol, n (%)	86 (21.1%)	85 (32.6%)	1 (0.7%)	0.000
SBP, mmHg	149.3 ± 24.6	146.2 ± 24.2	154.9 ± 24.5	0.001
DBP, mmHg	83.3 ± 13.7	82.9 ± 14.0	83.8 ± 13.2	0.533
CAD, n (%)	175 (42.9%)	132 (50.6%)	43 (29.3%)	0.000
Hypertension, n (%)	268 (65.7%)	160 (61.3%)	108 (73.5%)	0.013
DM, n (%)	97 (23.8%)	55 (21.1%)	42 (28.6%)	0.088
Hyperlipidaemia, n (%)	24 (5.9%)	16 (6.1%)	8 (5.4%)	0.777
Cerebral infarction, n (%)	183 (44.9%)	107 (41%)	76 (51.7%)	0.037
COPD, n (%)	15 (3.7%)	14 (5.4%)	1 (0.7%)	0.016
Carotid atherosclerosis, n (%)	251 (61.5%)	164 (62.8%)	87 (59.2%)	0.467
Carotid artery stenosis, n (%)	50 (12.3%)	35 (13.4%)	15 (10.2%)	0.344
Aortic sclerosis, n (%)	146 (35.8%)	101 (38.7%)	45 (30.6%)	0.102
CIMT, mm	1.50 (0.5–6.7)	1.70 (0.6–6.7)	1.30 (0.5–5.0)	0.000
Triglyceride, mmol/L	1.47 (0.35–8.87)	1.35 (0.43–8.87)	1.6 (0.35–8.51)	0.010
TC, mmol/L	4.40 (0.52–8.58)	4.25 (0.52–7.95)	4.62 (2.34–8.58)	0.000
HDL-C, mmol/L	1.19 (0.54–2.40)	1.15 (0.64–2.32)	1.26 (0.54–2.40)	0.000
LDL-C, mmol/L	2.49 (0.87–6.47)	2.41 (0.87–5.81)	2.66 (1.01–6.47)	0.003
Lipoprotein (a), mg/L	194 (15–1398)	186 (24–1214)	213 (15–1398)	0.889
CRP, mg/L	4.1 (1–189)	4.3 (1–164)	3.4 (1–189)	0.254
WBC count	6.6 (3–19.2)	6.63 (3–19.2)	6.4 (3.6–17.3)	0.174
Platelet count	207 (68–576)	193 (68–424)	234 (81–576)	0.000
RBC	4.4 (2.1–15.9)	4.5 (2.1–15.9)	4.2 (3.07–5.65)	0.000
Hemoglobin, g/L	132 (66–188)	138 (66–188)	126 (85–152)	0.000
Statin agent, n (%)	103 (25.2%)	67 (25.7%)	36 (24.5%)	0.792
Anti-platelets agent, n (%)	222 (54.4%)	150 (57.5%)	72 (49%)	0.099
Anti-hypertension agent, n (%)	194 (47.5%)	118 (45.2%)	76 (51.7%)	0.208
Hypoglycemic agent, n (%)	56 (13.7%)	29 (11.1%)	27 (18.4%)	0.041

Statistical differences: *P* < 0.05

*BSA* body surface area, *SBP* systolic blood pressure, *DBP* diastolic blood pressure, *CAD* coronary artery disease, *DM* diabetes mellitus, *COPD* chronic obstructive pulmonary disease, *CIMT* carotid intima-media thickness, *TC* total cholesterol, *HDL-C* high-density lipoprotein cholesterol, *LDL-C* low-density lipoprotein cholesterol, *CRP* C-reaction protein, *WBC* white blood cell

**Table 2:** Aortic diameter of each segment

Aortic diameter	All Mean ± SD or median or n (%)	Male Mean ± SD or median or n (%)	Female Mean ± SD or median or n (%)	*P* value
Anulus	19.3 (15.6–24.7)	19.9 (16.2–24.7)	18.4 (15.6–22.4)	0.000
Sinuses of Valsalva	32.1 (24.7–42.0)	33.4 (25.8–42.0)	30.1 (24.7–38.6)	0.000
Sinotubular junction	25.1 (19.0–34.3)	25.8 (19–34.3)	23.9 (19.3–29.6)	0.000
Proximal arch	28.7 ± 2.4	29.0 ± 2.41	28.1 ± 2.3	0.000
Mid arch	23.4 ± 2.3	23.8 ± 2.3	22.7 ± 2.1	0.000
Distal arch	20.1 ± 1.9	20.5 ± 1.8	19.6 ± 1.8	0.000
Ascending aorta	32.0 (24.8–42.3)	32.2 (25.1–42.3)	31.8 (24.8–42.1)	0.078
Descending aorta	19.5 ± 2.1	19.9 ± 2.1	18.7 ± 1.9	0.000
Abdominal aorta	15.2 ± 1.7	15.6 ± 1.7	14.5 ± 1.4	0.000

Statistical differences: *p* < 0.05

### Aortic diameter and cardiovascular risk factors

As displayed in Tables 3, 4 and 5, univariate analysis (Table 3) suggested that the diameters of the annulus, sinuses of Valsalva and sinotubular junction were positively correlated with sex, BSA, smoking, drinking, DBP, RBC count and hemoglobin. Among them only annulus diameter was negatively correlated with age and DM. As for aortic arch segments (Table 4), the diameter of all three segmental arches were positively correlated with sex, BSA, smoking, DBP, RBC count and hemoglobin, and were negatively correlated hypoglycemic agents use. Unlike the adjacent segments, ascending aorta diameter was positively correlated with age, BSA, SBP, DBP, hypertension, hemoglobin and antihypertension agents use, and negatively correlated with statin agents use. The diameter of descending aorta and abdominal aorta had some common influencing factors (Table 5). Positively related factors included sex, BSA, smoking, drinking, CIMT and hemoglobin. Aortic diameters of all nine segments were significantly negatively related to the use of hypoglycemic agent.

**Table 3:** Correlation between the aortic diameters of annulus, sinuses of Valsalva, sinotubular junction and risk factors by univariate regression analysis

Variables	Annulus		Sinuses of Valsalva		Sinotubular junction	
	Coefficient ρ	*P* values	Coefficient ρ	*P* values	Coefficient ρ	*P* values
Sex	1.487	0.000	3.257	0.000	2.054	0.000
Age, year	− 0.031	0.000	− 0.002	0.900	− 0.024	0.050
BSA	4.340	0.000	8.596	0.000	5.707	0.000
Smoking	1.115	0.000	2.033	0.000	1.279	0.000
Drinking	0.601	0.002	1.549	0.000	0.754	0.019
SBP	− 0.001	0.841	0.014	0.038	0.005	0.361
DBP	0.011	0.049	0.032	0.009	0.027	0.004
Hypertension	− 0.254	0.124	0.087	0.808	0.075	0.788
DM	− 0.471	0.010	− 0.740	0.063	− 0.364	0.248
CAD	0.146	0.357	− 0.067	0.833	− 0.407	0.189
Hyperlipidaemia	− 0.168	0.614	− 0.152	0.833	− 0.370	0.510
Triglyceride, mmol/L	0.032	0.607	− 0.069	0.610	0.086	0.410
TC, mmol/L	0.020	0.780	− 0.171	0.259	− 0.205	0.081
HDL-C, mmol/L	− 0.146	0.632	− 0.222	0.736	0.109	0.832
LDL-C, mmol/L	0.001	0.987	− 0.165	0.399	− 0.230	0.131
Lipoprotein (a)	− 0.001	0.084	− 0.001	0.324	− 0.001	0.269
COPD	–	–	1.555	0.084	–	–
Arteriosclerosis	− 0.274	0.094	–	–	–	–
RBC	0.319	0.002	0.461	0.036	0.340	0.047
Hemoglobin	0.030	0.000	0.065	0.000	0.043	0.000
Statin agent	− 0.141	0.435	− 0.100	0.797	− 0.388	0.201
Hypoglycemic agent	− 0.784	0.001	− 1.551	0.002	− 0.915	0.017
Anti-hypertension agent	− 0.159	0.311	0.294	0.387	0.243	0.357

Statistical differences: *P* < 0.05; only baseline variables and other interested variables that showed a univariate relationship with aortic diameter with *P* < 0.1 were displayed in the table

*BSA* body surface area, *SBP* systolic blood pressure, *DBP* diastolic blood pressure, *CAD* coronary artery disease, *DM* diabetes mellitus, *COPD* chronic obstructive pulmonary disease, *TC* total cholesterol, *HDL-C* high-density lipoprotein cholesterol, *LDL-C* low-density lipoprotein cholesterol, *RBC* red blood cell

– Not applicable

**Table 4:** Correlation between the aortic diameters of aortic arch and risk factors by univariate regression analysis

Variables	Proximal arch		Mid arch		Distal arch	
	Coefficient ρ	*P* values	Coefficient ρ	*P* values	Coefficient ρ	*P* values
Sex	0.940	0.000	1.092	0.000	0.927	0.000
Age, year	0.006	0.592	0.004	0.718	0.012	0.152
BSA	2.816	0.000	2.796	0.000	1.860	0.002
Smoking	0.800	0.001	0.631	0.006	0.457	0.015
Alcohol	0.714	0.015	0.359	0.191	0.520	0.021
SBP	0.013	0.007	0.007	0.118	0.010	0.010
DBP	0.030	0.000	0.037	0.000	0.033	0.000
Hypertension	0.771	0.002	0.605	0.010	0.373	0.055
DM	− 0.391	0.165	− 0.571	0.029	− 0.845	0.000
CAD	− 0.750	0.002	− 0.701	0.002	− 0.357	0.056
Hyperlipidaemia	0.591	0.247	− 0.057	0.905	0.134	0.733
Triglyceride	− 0.041	0.662	− 0.008	0.928	− 0.117	0.109
TC, mmol/L	− 0.028	0.792	− 0.134	0.179	− 0.042	0.615
HDL-C, mmol/L	− 0.271	0.560	0.115	0.790	0.268	0.455
LDL-C, mmol/L	0.045	0.747	− 0.232	0.071	− 0.030	0.779
Lipoprotein (a)	0.000	0.445	− 0.001	0.161	0.000	0.692
Cerebral infarction	–	–	0.405	0.072	–	–
Carotid atherosclerosis	–	–	–	–	0.339	0.075
RBC	0.421	0.007	0.407	0.005	0.288	0.016
Hemoglobin	0.029	0.000	0.027	0.000	0.017	0.004
Statin agent	− 0.502	0.069	− 0.392	0.128	− 0.056	0.793
Hypoglycemic agent	− 1.044	0.003	− 1.123	0.001	− 1.391	0.000
Anti-hypertension agent	0.576	0.016	0.447	0.046	0.328	0.076

Statistical differences: *P* < 0.05; only baseline variables and other interested variables that showed a univariate relationship with aortic diameter with *P* < 0.1 were displayed in the table

*BSA* body surface area, *SBP* systolic blood pressure, *DBP* diastolic blood pressure, *CAD* coronary artery disease, *DM* diabetes mellitus, *COPD* chronic obstructive pulmonary disease, *CIMT* carotid intima-media thickness, *TC* Total cholesterol, *HDL-C* high-density lipoprotein cholesterol, *LDL-C* low-density lipoprotein cholesterol, *RBC* red blood cell

– Not applicable

**Table 5:** Correlation between the aortic diameters of ascending aorta, descending aorta, abdominal aorta and risk factors by univariate regression analysis

Variables	Ascending aorta		Descending aorta		Abdominal aorta	
	Coefficient ρ	*P* values	Coefficient ρ	*P* values	Coefficient ρ	*P* values
Sex	0.664	0.055	1.135	0.000	1.100	0.000
Age, year	0.043	0.005	0.019	0.056	− 6.926E − 005	0.993
BSA	2.828	0.010	4.044	0.000	2.454	0.000
Smoking	0.230	0.499	0.755	0.000	0.675	0.000
Alcohol	0.393	0.335	0.607	0.019	0.727	0.000
SBP	0.022	0.001	0.009	0.045	0.003	0.383
DBP	0.051	0.000	0.025	0.001	0.012	0.054
Hypertension	1.204	0.001	0.631	0.005	0.271	0.126
DM	− 0.072	0.853	− 0.334	0.180	− 0.262	0.184
CAD	− 0.206	0.539	− 0.102	0.633	− 0.212	0.212
Hyperlipidaemia	0.561	0.427	− 0.128	0.777	0.241	0.501
Triglyceride, mmol/L	− 0.156	0.237	− 0.010	0.777	− 0.035	0.600
TC, mmol/L	− 0.024	0.872	− 0.051	0.593	− 0.051	0.500
HDL-C, mmol/L	1.090	0.090	− 0.484	0.239	0.516	0.113
LDL-C, mmol/L	0.042	0.828	− 0.017	0.892	− 0.052	0.591
Lipoprotein (a)	0.000	0.547	− 0.001	0.249	0.000	0.371
Carotid atherosclerosis	–	–	–	–	0.311	0.072
CIMT	–	–	0.252	0.018	0.190	0.024
CRP	–	–	− 0.010	0.015	–	–
WBC	–	–	− 0.086	0.050	–	–
Hemoglobin	0.024	0.029	0.027	0.000	0.022	0.000
Statin agent	− 0.832	0.029	− 0.269	0.271	− 0.119	0.537
Hypoglycemic agent	− 0.791	0.101	− 0.935	0.002	− 0.674	0.006
Anti-hypertension agent	0.982	0.003	0.515	0.015	0.232	0.167

Statistical differences: *P* < 0.05; only baseline variables and other interested variables that showed a univariate relationship with aortic diameter with *P* < 0.1 were displayed in the table

*BSA* body surface area, *SBP* systolic blood pressure, *DBP* diastolic blood pressure, *CAD* coronary artery disease, *DM* diabetes mellitus, *COPD* chronic obstructive pulmonary disease, *CIMT* carotid intima-media thickness, *TC* total cholesterol, *HDL-C* high-density lipoprotein cholesterol, *LDL-C* low-density lipoprotein cholesterol, *CRP* C-reaction protein, *WBC* white blood cell

– Not applicable

Inconsistent with the unadjusted regression, as showed in Tables 6, 7 and 8, multivariable-adjusted association revealed significant increases in diameters with age (*P* < 0.05) in segments of mid arch (β = 0.026, *P* = 0.024), distal arch (β = 0.021, *P* = 0.030), ascending aorta (β = 0.066, *P* < 0.001) and descending aorta (β = 0.039, *P* < 0.001). The diameter of annulus (β = 0.891, *P* < 0.001), sinuses of Valsalva (β = 2.128, *P* < 0.001), sinotubular junction (β = 1.320, *P* = 0.001), mid arch (β = 0.810, *P* = 0.016), distal arch (β = 0.734, *P* = 0.007) and abdominal aorta (β = 0.845, *P* = 0.001) were significantly increased in males. BSA was positively associated with the diameter of the annulus (β = 1.673, *P* = 0.008), sinuses of Valsalva (β = 3.793, *P* = 0.005), sinotubular junction (β = 2.253, *P* = 0.041), ascending aorta (β = 3.429, *P* = 0.018), descending aorta (β = 3.253, *P* < 0.001). Five of nine segments including sinotubular junction (β = 0.025, *P* = 0.039), mid arch (β = 0.046, *P* < 0.001), distal arch (β = 0.038, *P* < 0.001), ascending aorta (β = 0.064, *P* < 0.001) and descending aorta (β = 0.032, *P* = 0.001) were observed for the positive associations of DBP with aortic diameters. SBP was only observed for the inverse association with the mid arch diameter (β = -0.019, *P* = 0.002). Differently, hypertension was observed for its inverse association with the diameter of proximal arch (β = 0.803, *P* = 0.023), mid arch (β = 0.781, *P* = 0.016) and descending aorta (β = 0.661, *P* = 0.030). CAD had a significant reverse relationship with the diameter of transverse arch (β =  − 1.064, *P* < 0.001; β =  − 1.068, *P* < 0.001; β =  − 0.478, *P* = 0.017) and abdominal aorta (β =  − 0.489, *P* = 0.009). Lipid profile had no relation to any aortic segments in unadjusted regression, while HDL-C turned to have a significantly positive effect on the diameter of ascending (β = 1.348, *P* = 0.042) and abdominal aorta (β = 0.947, *P* = 0.004). The use of hypoglycemic drugs was independently negatively associated with the diameter of transverse arch (β =  − 1.105, *P* = 0.023; β =  − 0.954, *P* = 0.032; β =  − 0.919, *P* = 0.011), descending aorta (β =  − 0.923, *P* = 0.026) and abdominal aorta (β =  − 0.666, *P* = 0.047).

**Table 6:** Relationship between the aortic diameters of annulus, sinuses of Valsalva, sinotubular junction and risk factors by multivariate regression analysis

Variables	Annulus		Sinuses of Valsalva		Sinotubular	
	Coefficient β	*P* values	Coefficient β	*P* values	Coefficient β	*P* values
Sex	0.891	0.000	2.128	0.000	1.320	0.001
Age, year	− 0.019	0.014	–	–	_	_
BSA	1.673	0.008	3.793	0.005	2.253	0.041
DBP	_	_	_	_	0.026	0.032
Smoking	0.459	0.013	–	–	–	–
Hemoglobin	_	_	0.029	0.044	_	_

Statistical differences: *P* < 0.05; only significant variables and data were displayed in the table

*BSA* body surface area, *DBP* diastolic blood pressure

– Not applicable

**Table 7:** Relationship between the aortic diameters of aortic arch and risk factors by multivariate regression analysis

Variables	Proximal arch		Mid arch		Distal arch	
	Coefficient β	*P* values	Coefficient β	*P* values	Coefficient β	*P* values
Sex	–	–	0.810	0.016	0.734	0.007
Age, year	–	–	0.026	0.024	0.021	0.030
SBP, mmHg	_	_	− 0.019	0.002	_	_
DBP, mmHg	_	_	0.046	0.000	0.038	0.000
CAD	− 1.064	0.000	− 1.068	0.000	− 0.478	0.017
Hypertension	0.803	0.023	0.781	0.016	–	–
Hypoglycemic agent	− 1.105	0.023	− 0.954	0.032	− 0.919	0.011

Statistical differences: *P* < 0.05; only significant variables and data were displayed in the table

*SBP* systolic blood pressure, *DBP* diastolic blood pressure, *CAD* coronary artery disease

– Not applicable

**Table 8:** Relationship between the aortic diameters of ascending aorta, descending aorta, abdominal aorta and risk factors by multivariate regression analysis

Variables	Ascending aortaAscending aorta		Descending aorta		Abdominal aorta	
	Coefficient β	*P* values	Coefficient β	*P* values	Coefficient β	*P* values
Sex	–	–	_	_	0.845	0.001
Age, year	0.066	0.000	0.039	0.000	–	–
BSA	3.429	0.018	3.253	0.000	–	–
DBP, mmHg	0.064	0.000	0.032	0.001	–	–
CAD	–	–	–	–	− 0.489	0.009
Hypertension	_	_	0.661	0.030	_	_
HDL-C, mmol/L	1.348	0.042	_	_	0.947	0.004
Hypoglycemic agent	_	_	− 0.923	0.026	− 0.666	0.047

Statistical differences: *P* < 0.05; only significant variables and data were displayed in the table

*BSA* body surface area, *DBP* diastolic blood pressure, *CAD* coronary artery disease, *HDL-C* high-density lipoprotein cholesterol, *CRP* C-reaction protein

_ Not applicable

### Ascending aorta diameter with no dilation and cardiovascular risk factors

Ascending aortic dilation was considered in 38 patients with diameter greater than 37 mm. We conducted an additional multivariate linear regression excluding the participants with ascending aortic dilation, and found that age (β = 0.055, *P* < 0.001), BSA (β = 2.746, *P* = 0.030), and diastolic blood pressure (β = 0.034, *P* = 0.016) were independent positively associated with ascending aortic diameter with no dilation (Table 9).

**Table 9:** Relationship between risk factors and ascending aortic diameter with no dilation by multivariate linear regression analysis

Variables	Multivariate linear regression	
	Coefficient β	*P* value
Sex	− 0.083	0.847
Age	0.055	0.000
BSA	2.746	0.030
Smoking	− 0.346	0.355
Alcohol	0.002	0.997
SBP	0.000	0.987
DBP	0.034	0.016
CAD	0.020	0.951
Hypertension	0.705	0.093
DM	− 0.174	0.721
Hyperlipidaemia	− 0.181	0.779
Triglyceride, mmol/L	− 0.237	0.053
HDL-C, mmol/L	0.526	0.355
Lipoprotein (a), mg/L	0.000	0.731
Platelet count	− 0.004	0.069
Statin agent	− 0.392	0.220
Anti-hypertension agent	0.187	0.631
Hypoglycemic agent	− 0.407	0.492
Hemoglobin	0.013	0.256

Sex, age, BSA, smoking, drinking, SBP, DBP, hypertension, DM, CAD, Hyperlipidaemia, triglyceride, HDL-C, LP (a), statin agent, hypoglycemic agent and anti-hypertension agent were considered clinically relevant and were entered into multivariate linear regression analysis of ascending aorta diameter with no dilation, regardless of whether it is significantly related to aortic diameter. Other interested variables that showed a univariate relationship with aortic diameter with *P* < 0.1 were included

Statistical differences: *P* < 0.05

*BSA* body surface area, *SBP* systolic blood pressure, *DBP* diastolic blood pressure, *CAD* coronary artery disease, *DM* diabetes mellitus, *HDL-C* high-density lipoprotein cholesterol

## Discussion

The current research indicated that some cardiovascular risk factors such as HDL-C, SBP, DBP. BSA may have an effect on increasing the diameter of the aorta in a specific segment. Also, to explore the risk factors of ascending aorta expansion, we found that age, BSA and DBP were independent positively influencing factors of ascending aortic diameter with no dilation.

Aortic diameter is an important indicator of aortic dilation disease. According the studies focused on different aortic segments, there is an apparent overlap between the influencing factors of the aortic diameter and cardiovascular risk factors [12, 13, 15], which can also be reflected in our current research.

In agreement with the previous research [12, 16], the present study shows a link between aortic diameter enlargement and aging, sex and BSA in some aortic segments. The aorta size increases throughout life, accompanied by loss of compliance and increased wall stiffness, leading to arterial dilation. A population-based study of Germany, Hatemi et al. [12] found that the diameter of each segment of the thoracic aorta and abdominal aorta expands with age, but we failed to show a statistical relationship in the abdominal aorta. Sex difference in aortic diameter and the risk of aortic diseases is a well-known fact [17]. The current results suggested that the diameters of almost all aortic segments were higher in males except for ascending aorta (*P* = 0.078). However, our multivariable model did not find an independent association between sex and the diameter of ascending aorta, descending aorta and proximal arch. At the same time, the confirmatory association of aortic diameter with BSA was only reflected in thoracic aorta segments in current results, supported by prior studies[16, 17].

Previous studies reported contradictory results about the role of high blood pressure as a major risk factor for aortic dilation. In a general population [12], DBP was positively and SBP was reversely associated with thoracic and abdominal aortic diameters. Glauser et al. [10] and Vasan et al. [18] both reported a completely consistent view in aortic root and abdominal aorta. However, in a cardiovascular risk screening program conducted in Paris [19], ascending aortic diameter was positively associated with SBP and DBP after excluding participants taking antihypertensive medication. In the current study, blood pressure indexes were evaluated at nine sites with small size samples. Our data indicated that DBP was positively associated with the thoracic aorta diameter at several sites but not with abdominal aorta diameter, while SBP was only negatively associated with the diameter of the mid arch. Also, multivariate linear regression analysis excluding aortic dilation confirmed the independent positive association of DBP to ascending aortic expansion. Unlike the limited effects of blood pressure on specific sites (beta =  − 0.019 to 0.064), hypertension had considerate effect on aortic diameter of proximal arch, mid arch and descending aorta in our results. Previous studies have observed an independent and significant relationship between hypertension and the increase in the diameter of the abdominal aorta [15, 20], but rare study has found the effect of hypertension on the diameter of each segment of the thoracic aorta. Contribution of hypertension and blood pressure in aortic diameter enlargement remains unclear at present. Follow-up studies need to be carried out.

Several studies have found a dose–response relationship between smoking intensity and the risk of AAA [21, 22]. One epidemiological study indicates that smoking history is the strongest factor associated with AAA progression [23]. Mechanistic research report that smoking promotes the degradation of collagen and elastin and consequent weakening of the arterial wall by highly expressed matrix metalloproteinase in aortic wall, finally leading to aortic aneurysm formation [24]. Although we observed that smoking had a robust association with the diameter of almost all segments apart from ascending aorta in the unadjusted model, the strength of this association could not be further demonstrated by fully adjusted analysis. Only the annulus diameter showed its independent association with smoking. The lack of association with abdominal aorta diameter was consistent with previous studies [7, 25]. Contrarily, some studies indicated that incremental widening of the abdominal and ascending aorta was independently related to smoking [6, 20]. Smoking cessation may have a certain effect on alleviating the dilation of the aorta.

In an unadjusted model, serum lipids were not relevant to aorta diameter of any segment. Surprisingly, HDL-C were an independent determinant of the diameters of ascending and abdominal aorta. Although previous studies on the relation among AAAs, abdominal aortic diameters and serum lipid levels were contradictory [7, 25]. Dyslipidemia has grown in importance among well-established risk factors for AAAs. A prospective study cohort for AAA patients found that HDL-C predicted the growth rate of aneurysms for its inverse association with AAA size [26]. Wang et al. [7] found that LDL-C and ratios of TC/HDL-C and LDL-C/HDL-C were independent negative determinants of infrarenal aortic diameter, and infrarenal aortic diameter was significantly positively associated with HDL-C (r = 0.139, *P* = 0.006) independent of age, sex, and height. The current research held the consistent view. Based on the results, we could speculate that HDL-C may have an effect on the increase in the diameter of the ascending aorta and abdominal aorta. The use of statins and the small research samples may affect the accuracy of the outcome. The effect of serum lipids on aortic dilation needs further study.

In contrast, diabetes mellitus plays a protective role in dilated aortic diseases [8, 27], characterized by the accumulation of collagen in the aortic wall and subsequent increases in matrix volume [28]. Interestingly, a rat study found that the total count of elastic fibers, fragmentation of the elastic lamina, pericellular matrix deposition, and cell loss/substitution in the tunica media were higher in the diabetic + smoker group (DSG) aorta than those in the smoker group (SG) aorta [24].The negative relationship between the presence of DM and aortic diameter was supported by a few other reports [8, 15]. However, we found only that DM was inversely related with the diameters of annulus, mid and distal arch in univariate analysis. Consistently, there was an independent negative correlation between the use of hypoglycemic drugs and the aorta diameter of some segments. The effect of blood sugar control on the diameter of the aorta needs further investigation.

Atherosclerosis represents an important independent risk factor for AAA formation [9]. In the Tromsø Study [20], as the measures of subclinical atherosclerosis, coronary artery calcium burden but not CIMT were independently associated with larger aortic diameter, which was supported by a population-based follow-up study [11]. In the current unadjusted analysis, CIMT was only positively associated with descending and abdominal aorta diameter, but was not an independent indicator of any segment. Carotid atherosclerosis, carotid artery stenosis and aortic sclerosis represented no association to all segments' diameters. Differently, coronary artery disease represented a significant independent relationship with the diameter of transverse arch and abdominal aorta in the current multivariate analysis. However, whether this association between atherosclerosis and aortic aneurysm is causal or a result of common shared risk profiles remains unknown. Johnsen et al. [29] indicated that no dose–response relationship between abdominal aortic diameter and atherosclerosis burden assessed as carotid total plaque area, common femoral lumen diameter, and self-reported coronary heart disease, suggested that atherosclerosis may not be a causal event in AAA, but occurred concurrently with aneurysm expansion or secondary to aneurysm expansion.

The segmental inconsistency may be ascribed to distinct structural, genetic, and biochemical factors [1, 30]. Specific segments of the thoracic and abdominal aorta have differences in vascular mechanics, atherosclerotic plaque deposition, MMPs distribution, and cell signaling pathways, which may lead to differences in each segment's susceptibility to risk factors [30].

### Limitation

First, the current study was an observational study in which potential confounders and selection bias could not be fully adjusted. Second, potential misunderstanding due to missing data on use of over-the-counter medication has to be taken into account. Third, the sample size was not large enough and all individuals were included non-randomly resulting in reduced power of the test. Lastly, the current research was biased toward patients with cardiovascular diseases so that some findings may have limited generalizability to non-cardiovascular diseases patients with cardiovascular risk factors. The listed limitations need to be solved by well-designed large observational cohort studies.

## Conclusion

In conclusion, different segments of aortic diameter may have different independent related factors. Aortic segmental diameters were associated with DBP, HDL-C, atherosclerosis and other traditional cardiovascular risk factors. These findings may provide new information for understanding the potential mechanism of the early stages of aortic dilation. Additionally, the methods of exploring novel biomarkers for the risk prediction, prevention and early diagnosis of aortic dilation diseases should take segment specificity into consideration. The well-designed studies including cohort studies and molecular level research need further development.

## Data Availability

The datasets used and/or analyzed during the current study are available from the corresponding author on reasonable request.

## Abbreviations

AAA: Abdominal aortic aneurysm
BSA: Body surface area
TC: Total cholesterol
HDL-C: High-density lipoprotein cholesterol
LDL-C: Low-density lipoprotein cholesterol
CRP: C-reactive protein
WBC: White blood cell
RBC: Red blood cell
CTMT: Carotid intima-media thickness
DM: Diabetes mellitus
SBP: Systolic blood pressure
DBP: Diastolic blood pressure
CAD: Coronary artery disease
COPD: Chronic obstructive pulmonary disease
VIF: Variance inflation factor
